# Near-Infrared Hyperspectral Imaging as a Monitoring Tool for On-Demand Manufacturing of Inkjet-Printed Formulations

**DOI:** 10.1208/s12249-021-02091-x

**Published:** 2021-08-10

**Authors:** Sandra Stranzinger, Matthias Wolfgang, Emma Klotz, Otto Scheibelhofer, Patrizia Ghiotti, Johannes G. Khinast, Wen-Kai Hsiao, Amrit Paudel

**Affiliations:** 1 Research Center Pharmaceutical Engineering (RCPE) GmbH, Inffeldgasse 13, 8010, Graz, Austria; 2 Graz University of Technology, Institute of Medical Engineering, Stremayrgasse 16, 8010, Graz, Austria; 3 UCB Pharma S.A., Allée de la Recherche 60, 1070 Brussels, Belgium; 4 Graz University of Technology, Institute for Process and Particle Engineering, Inffeldgasse 13, 8010, Graz, Austria

**Keywords:** Near-infrared hyperspectral imaging (NIR-HSI), Inkjet technology, Predictive models, Process Analytical Technology (PAT), Personalized medicine

## Abstract

This study evaluates the potential use of near-infrared hyperspectral imaging (NIR-HSI) for quantitative determination of the drug amount in inkjet-printed dosage forms. We chose metformin hydrochloride as a model active pharmaceutical ingredient (API) and printed it onto gelatin films using a piezoelectric inkjet printing system. An industry-ready NIR-HSI sensor combined with a motorized movable linear stage was applied for spectral acquisition. Initial API-substrate screening revealed best printing results for gelatin films with TiO_2_ filling. For calibration of the NIR-HSI system, escalating drug doses were printed on the substrate. After spectral pre-treatments, including standard normal variate (SNV) and Savitzky-Golay filtering for noise reduction and enhancement of spectral features, principal component analysis (PCA) and partial least squares (PLS) regression were applied to create predictive models for the quantification of independent printed metformin hydrochloride samples. It could be shown that the concentration distribution maps provided by the developed HSI models were capable of clustering and predicting the drug dose in the formulations. HSI model prediction showed significant better correlation to the reference (HPLC) compared to on-board monitoring of dispensed volume of the printer. Overall, the results emphasize the capability of NIR-HSI as a fast and non-destructive method for the quantification and quality control of the deposited API in drug-printing applications.

**Graphical abstract:**

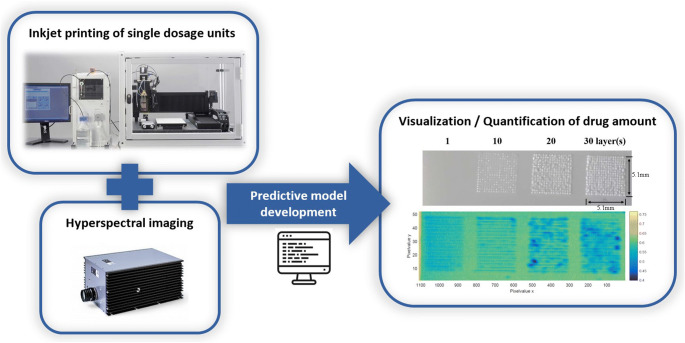

## Introduction

Current pharmaceutical research exhibits increasing interest in printing technology, since it combines dosing strength flexibility with accuracy and precision of established printing technologies, making it a tempting tool for personalized medicine. The possibility of on-demand manufacturing and real-time release of a dosage form could accelerate clinical trial supply, especially in adaptive clinical trials during which the dose allocation may change as a result of the open clinical protocol. In particular, the need for personalized and accurate drug dosing, as well as improved dose compliance and adherence, has created a unique opportunity for inkjet technology in the field of pharmaceutics (1–4). Daly et al. recently published a comprehensive review on the pharmaceutical application of inkjet printing technologies discussing about current technologies and future challenges (5).

Inkjet printing offers potential for accurate, consistent, spatially localized, low-cost, and high-speed dispensing of a large variety of multicomponent systems containing one or several active pharmaceutical ingredients (APIs) and excipients in a microarray format which makes it possible to achieve uniform distribution of the constituents (6). Moreover, the possibility to print on many porous or non-porous substrates and biodegradable films has opened a new perspective for on-demand pharmaceutical manufacturing of personalized oral solid dosage forms (7, 8). For inkjet printing, an introduction of a regulatory pathway that permits the development and use of personalized medical products via inkjet printing is required (9), also with regard to standardizing the characterization and quality control (10).

Implementing process monitoring in inkjet printing process enables the monitoring of deposited drug of each dosage unit, tailored for a specific patient. Dosing in the final print is a function of the API concentration in the ink solution used for printing and the total volume of droplets deposited per dosage unit. The ability to monitor and control the content uniformity and compositional homogeneity in the printed dosage forms is therefore crucial (11). Since conventional chromatographic methods for analyzing API content of printed samples are laborious, destructive, and time-consuming, alternative methods for quality control have to be developed. On-line analysis of printed samples without manipulating or treating the sample further would offer a more pragmatic way of determining and understanding the API distribution and homogeneity in various API-substrate combinations. From the industrial QC perspective, the quality of any printed sample should be monitored throughout the process, which would guarantee “100% in spec” high-quality end products.

Reliable and robust methods for QC are required to ensure the printed product targeted for personalized medicine, e.g., in point-of-care (PoC) applications. Edinger et al. comprehensively reviewed non-destructive analytical methods used for evaluation of printed dosage forms (12). In their review, Raman spectroscopy and chemical imaging are discussed as powerful tools for visualization and non-destructive quantification of inkjet-printed pharmaceuticals. The key prerequisite for using Raman is a strong Raman active API printed onto the Raman inactive excipient surface. The strong Raman scattering substrate reduces the accuracy of visualization and/or quantification of the printed API (13). Also, Raman spectroscopy is inherently prone to the fluorescence artifact (14). Further aspects to be considered are the porosity of the substrate and the penetration depth of the ink.

Colorimetry is reported as another promising quality control tool for individual inkjet-printed pediatric formulations (15). However, some limitations of this method have been discussed by Edinger et al: to name some, the color variations in API and substrate due to different manufacturers or color saturation (12).

Near-infrared spectroscopy (NIRS) and near-infrared chemical imaging (NIR-CI) have been applied in quantitative studies of printed dosage forms as well. During the last decade, a growing interest in the use of NIR-CI in the field of pharmaceutical analysis has been observed (16). Further, noteworthy reviews on the merits of NIRS with special emphasis on pharmaceutics and dosage forms are the reviews by De Beer et al. (17) and, more recent, the review by Stranzinger et al. (18). NIRS has been successfully been used for raw material testing, process monitoring, and product quality control (19–23). Also in the context of inkjet printing, NIRS is being widely adopted as a QC tool (9). NIRS further offers key advantages, like simple operation, rapid and destruction-free measurements within seconds, and applicability in different environments such as industry or laboratories. Further, the assets of NIRS are minimal effort for sample preparation and the possibility of scanning multiple spectra on the same object at once, obtaining more representative sample composition information.

Especially, the ability to provide repeatable, qualitative, and quantitative measurements within seconds makes near-infrared hyperspectral imaging (NIR-HSI) an attractive tool for QC of printed dosage forms. Specifically, using NIR-HSI, a multitude of spectra can be collected in fractions of a second and spatially resolved information about the nature and quantity of chemical components present can be obtained. Being a rapid and contact-free method, NIR-HSI can be integrated into continuous processes to allow in-line monitoring of the API distribution, the homogeneity of each dosage unit, and the overall consistency of final products. Further, NIR-HSI has already been employed in many stages along the pharmaceutical product life cycle, from early research and development to the final product analysis (24–27) and is therefore accepted more easily by the industry. In addition, NIR-HSI can provide a close monitoring of 2D-printed medicines in real time since no sample preparation is required prior to data acquisition and it provides opportunities for faster qualitative and quantitative measurements of solid dosage forms compared to the traditional spectroscopic and chromatographic techniques (28). Previous studies like, e.g., Vakili et al. have demonstrated that NIR-HSI is a reliable, rapid, and non-destructive method of optimizing quality control of planar-printed dosage forms (1). However, their studies dealt only with commercial, uncoated copying paper over-printed with ethanol-based ink solutions or very small areas under investigation (i.e., a circle with a diameter of 1 mm) using a compact handheld high-performance NIR reflection spectral device (19). We aim to extend their first proof of concept to actual ingestible substrates and industry-ready NIR-HSI equipment using water-based ink, which is a challenge to current printing systems due to the elevated surface tensions and viscosity. Further, we propose an alternative Raman-based spectral calibration approach, discussing critical steps to be considered during model development to guarantee predictive strength of the models. Moreover, one key aspect considered in our study is the visualization of the printed drug dose in the formulations by means of concentration distribution maps via HSI models.

## Materials And Methods

### Ink Solution

Due to its good water solubility and considerations on operational safety, we used metformin hydrochloride, a common orally administrated and well-known antidiabetic drug, as a model substance for 2D inkjet printing. Metformin hydrochloride, in its pure state, is a white, crystalline powder with a molecular weight of 165.62 g/mol, water solubility of 350 g/l at 20°C, and a pH of 6.7 at 20°C (for 10 g/l). The compound is only slightly soluble in ethanol and nearly insoluble in acetone and methylene chloride.

In order to prepare the required ink, 250 mg/ml of metformin hydrochloride (Sigma Aldrich, St. Louis, MO, USA) was dissolved in purified water (Milli-Q). The ink solution was filtered using a 0.45 μm Nylon syringe filter (Roth GmbH, Karlsruhe, Germany) prior to all printing experiments to decrease the risk of nozzle blockage.

### Printing Substrates

In preparation to the experimental main part of this study, six common excipients approved for pharmaceutical use were screened for their suitability as printing substrates to find the best API-substrate combination for chemometric model development. The commercially available and ready-to-use substrates used in the present study are listed below, with their main component(s) of the substrates in parentheses and in italic:

- Wafer paper sheets (Print4you Cake Toppers, UK) (*starch*)
- Ethylene vinyl acetate 9 (Versalis, San Donato Milanese, Italy) *(ethylene vinyl acetate—EVA)*
- Gelatin film strips (Capsugel, Bornem, Belgium) *(hard gelatin capsule matrix—HGC)*
- Gelatin film strips with 2% titanium dioxide (Capsugel, Bornem, Belgium) *(hard gelatin capsule matrix doped with titanium dioxide—HGC+TiO*_*2*_*)*
- Hydroxypropyl-methylcellulose (Capsugel, Bornem, Belgium) ***(****hydroxypropyl-methylcellulose—HPMC)*
- Edible icing sheets (DECO Enterprises Ltd, Sutton Valence, UK) *(corn starch/corn syrup—icing)*

All substrates are human-ingestible and are therefore considered suitable for orodispersible drug use.

### Piezoelectric Inkjet Printing (IJP)

In this study, a sciFLEXARRAYER S3 printer equipped with a sciDROPPICO print head (Scienion AG, Berlin, Germany) was used. This is an automated piezoelectric contact-free print system for research and production applications, which is capable of dispensing 30–800 pl per drop with a precision < 5 pl and accuracy < 15 pl according to the manufacturer. The system has a tele-centric video camera to examine the jetted drops for method optimization. The droplet’s size and shape can be influenced by changing the printing parameters, such as voltage, pulse length, and frequency for a given ink used. The printing software is capable of calculating the drop volume in picoliters based on dynamic image analysis. Drop optimization for metformin hydrochloride was achieved by setting the voltage to 90 V with a pulse length of 55 ms in all experiments. Mean droplet volume was calculated by dispersing 10,000 drops while measuring the volume of every 500th drop. A mean drop volume of 415 pl was calculated based on acquired images from the system camera.

### Printing Pattern for Calibration and Test Samples

**Figure 1:**
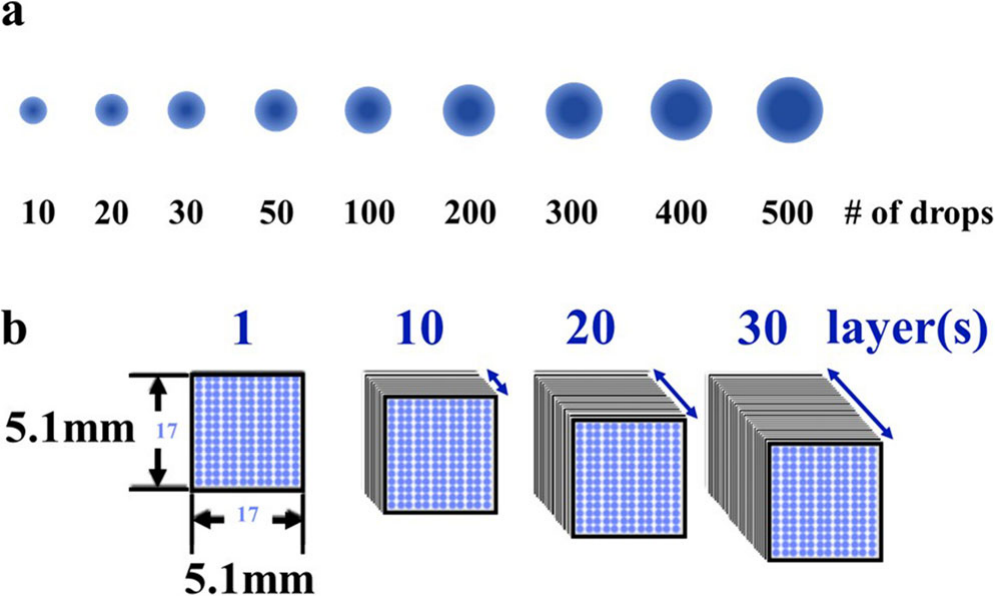
**a** Printing pattern schematic of calibration samples, 9 spots with an increasing number of drops per spot, ranging from 10 to 500 drops, and **b** printing pattern schematic of multilayer test samples

Based on the outcome of the printing substrate screening, calibration samples were printed on gelatin film strips with 2% titanium dioxide (*HGC+TiO*_*2*_), consisting of 9 spots in the *x*-direction. The number of drops was increased with each new spot (10, 20, 30, 50, 100, 200, 300, 400, and 500) in order to correlate the concentration in the sample to the NIR signal intensity like illustrated in Figure 1a. Additional square-shaped test patterns were printed to test the multilayer printing behavior. For this purpose, a matrix of 17 × 17 drops spaced at 300 μm in both directions was printed. This pattern was repeated in various layers, as depicted in Figure 1b.

### NIR-HSI Measurements

Hyperspectral imaging is a spatially resolved spectroscopic method which overcomes the limitations of common single-spot spectral analysis when it comes to heterogeneous samples. Datasets obtained via spectroscopic imaging are typically acquired as data cuboids, with the *x*- and *y*-dimensions covering a certain area of the sample (spatial information) and the z-dimension covering the corresponding spectral information. In our study, we used an industry-approved NIR-HSI sensor “Helios G2-320 Class” (EVK DI Kerschhaggl GmbH, Raaba, Austria) throughout all experiments. This sensor covers a spectral range of 930–1700 nm at 320 spatial positions simultaneously along a 38 mm long field of view (covered area is 38 × 0.119 mm^2^). The system works as a push-broom imager, requiring the sensor or the sample being moved, resulting in an infinite stream of samples under test. The required diffuse illumination was achieved by a fiber-optic ring light guide (Edmund Optics GmbH, York, UK) mounted co-axial to the object lens of the sensor, coupled with a tungsten-halogen lamp (Polytec GmbH, Waldbronn, Germany). Illumination was optimized to operate at low power in order to prevent drying effects and sample degradation, as well as to prevent interferences with the printer setup (print heads and ink are sensitive to temperature). The acquisition rate of the NIR-HSI sensor was set to 111 fps (corresponding to 9 ms exposure time for the sensor) throughout all experiments, to account for the given illumination in order to guarantee the best signal-to-noise ratio (S/N ratio) for data acquisition. It is noteworthy to mention that higher frame rates are possible by using increased illumination power — the successor of the used sensor can operate at up to 447 fps in full frame acquisition and is able to achieve even higher frame rates with reduced spectral coverage.

**Figure 2:**
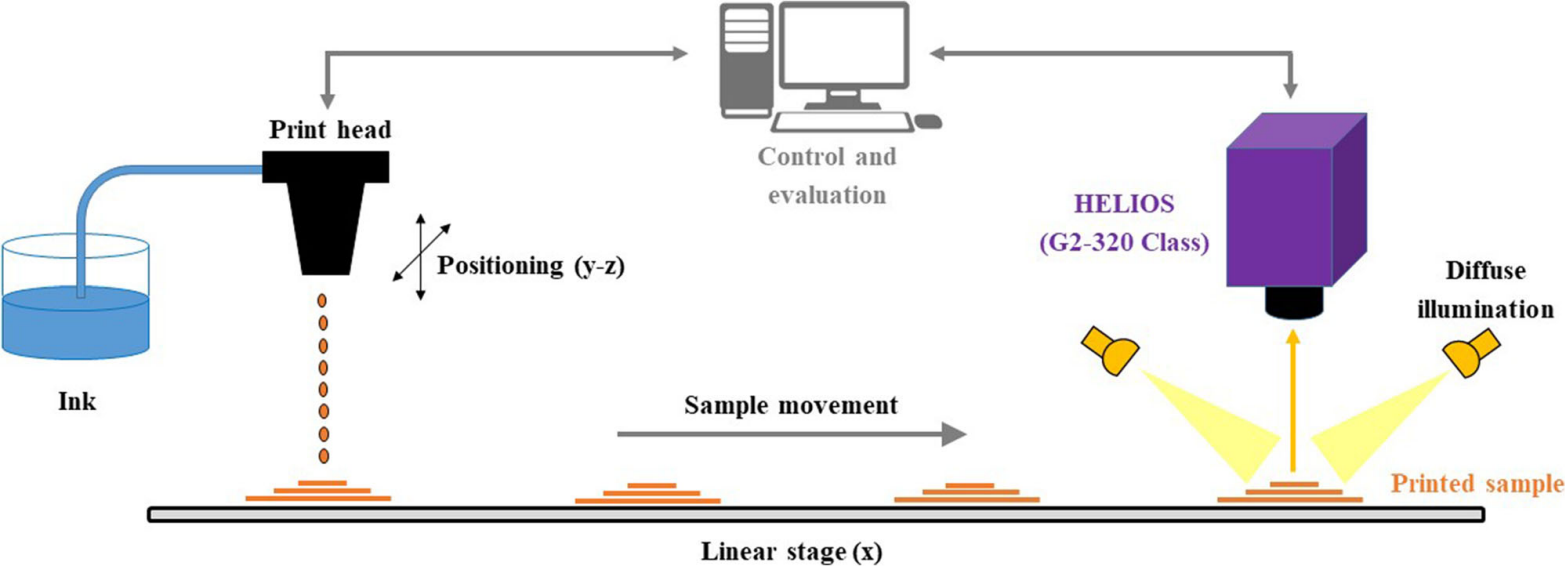
Schematic representation of experimental setup for NIR hyperspectral imaging measurements

Samples were placed on black anodized aluminum and subsequently moved using a linear stage (MOVTEC Wacht GmbH, Pforzheim, Germany) at a rate of 200 μm/s while being measured at 111 lines per second (=HSI-frames) with a sensor exposure time of 9 ms. A schematic representation of the experimental setup is shown in Figure 2. All spectral data acquisition was performed in reflectance mode. White and black reference spectra were acquired prior to measuring the samples using same system settings by acquiring the spectra of a Teflon sheet (white balance) and covering the lens (black balance).

#### Determination of Spatial and Temporal Resolution of the Used NIR-HSI Sensor

To determine the spatial resolution of the system, i.e., the resolution in the direction perpendicular to the moving linear stage (*y*-axis in all results), a “1951 US Air Force (USAF) resolution test chart” was used. The target was measured without using the linear stage and at the same sensor settings as all subsequent measurements. The resolution measurements were averaged and corrected based on white and black reference spectrum to reduce noise effects. No further processing or smoothing steps were undertaken. Spatial resolution was determined based on various line patterns of the test chart to be 0.114 mm/px at a 6σ criterion.

To determine the resolution in direction of the moving linear stage (*x*-axis in all results), as a first step, the point spread function of the system has to be calculated. For this purpose, the NIR-HSI sensor was set to an acquisition rate of 111 fps (as for all sample measurements) and a printed reference line with a width of 285 μm was moved below the sensor at a speed of 200 μm/s. The width of the reference line (*b*_line_) was confirmed based on independent measurements using a Senterra optical microscope (Bruker, Billerica, USA). The assumption was made that for the real line (*f*_line_), the systems point spread function (*f*_PSF_) and the measured line (*f*_meas_) can be displayed as a convolution of Gaussian functions: *f*_meas_ = *f*_line_ ∗ *f*_PSF_. This allows the line width to be expressed by the standard deviation (σ) of a Gaussian function, based on the rules of convolution:

1 $$ {\upsigma}_{PSF}=\sqrt{{\upsigma^2}_{meas}-{\upsigma^2}_{line}} $$

Thereby, it is possible to deduct the point spread function. To obtain σ_*meas*_, the number of pixels at full width half maximum (FWHM) *n*_FWHM_ was counted and used to calculate FWHM following the relationship:

2 $$ \mathrm{FWHM}=2\ \sqrt{2\ln 2}{\upsigma}_{\mathrm{meas}} $$

For the optical width *b*_*line*_, the assumption was made that the measured line width contains 6σ of the line’s Gaussian function due to the steep borders in the image. To convert the width in pixels into mm, a factor has to be determined by the frequency of measurement (*f*) and the speed of the linear stage (*v*) to be *k* = *v*/*f*. Via substitution, we can obtain the following relation:

3 $$ {\upsigma}_{PSF}=\sqrt{{\left(\frac{n_{FWHM}}{2\sqrt{\ 2\ln (2)\ }}k\right)}^2-{\left(\frac{b_{line}}{6}\right)}^2} $$

*n*_*FWHM*_ was determined to be 19 (pixels), *k* equals 20 μm and, as mentioned above, *b*_line_ was measured to be 285 μm, leading to a σ_PSF_ of 0.154 mm/px, also relying on a 6σ criterion.

### NIR Data Pre-treatment and Modeling

In multivariate analysis, pre-treatment of raw spectral data is required in order to extract the relevant spectral features and create consistent and reliable predictive models. In this study, the obtained NIR spectra were processed using MATLAB (MathWorks, Natick Massachusetts, USA). Absorption units were calculated using mean white and black reference spectra. By plotting spectral absorption as a function of spatial dimensions, the region of interest (ROI) for the relevant spectral information could be determined within the data cuboids. Based on these “streamlined” datasets, significantly more efficient calculations can be performed. Partial least squares (PLS) regression, as developed by Wold et al. (29), was executed on these datasets in order to create predictive models based on the filtered and numerical differentiated data. In the present study, the “plsregress” function of MATLAB which uses the SIMPLS algorithm was applied (30).

### Raman Chemical Imaging

In order to have a reference with regard to API content and API distribution per spot for quantitative model development, Raman spectroscopic measurements were performed. In contrast to previous studies like that of Edinger et al., which focuses on the direct comparison between Raman and NIRS for quantification of the printed API (31), we used Raman mapping to visualize the distribution of API loading within the printed calibration samples (shown in Figure 1a). Specifically, this was to determine whether the API concentration is more pronounced in certain areas of the printed pattern, highlighting the gradients of API concentration along the areas covered by the printed drops. Raman chemical imaging was executed using RamanStation 400F (PerkinElmer, Waltham, US), equipped with a 350 mW near-infrared laser operating at 785 nm as excitation source, capable of measuring a Raman shift range of 95–3500 cm^-1^.

Samples were positioned on a coordination stage and subsequently moved below the laser by using the proprietary software “Spectrum.” During the measurement, an area of 1 × 1 mm with a spacing of 100 μm (100 measurements per drop) was mapped with two replicates (3 complete scans in total) at an exposure time of 6 s, resulting in a total time for acquisition of 30 min per spot. For data analysis, the obtained Raman spectra were imported into MATLAB (MathWorks, Natick Massachusetts, USA) using the “fsmload” function (32). Since preliminary tests revealed that the peak at 735 cm^-1^ corresponds to metformin hydrochloride, the wave number range of 730–740 cm^-1^ was used for API concentration determination.

### High-Performance Liquid Chromatography (HPLC)

High-performance liquid chromatography (HPLC), widely used in quality control of raw materials and final dosage forms, was chosen as a reference method for a comparison between the concentrations predicted by the HSI models and the actual API content in the printed samples. In order to extract the API for further analysis, the printed samples were dissolved in 5 ml of deionized water (Milli-Q) and shaken at room temperature at 300 rpm for 2 h. The resulting API solution was analyzed using a Waters Alliance (Milford, USA) HPLC system.

Separation was carried out in a Zorbax SB-CN column (150 mm × 2.1 mm, 5 μm; Agilent Technologies, California, USA) using isocratic elution at a flow rate of 0.4 mL/min. The column temperature was set to 30°C and the auto sampler temperature was 20°C. The mobile phase consisted of a mixture of 90% 1 mM ammonium acetate in water (Milli-Q) and 10% acetonitrile. UV-detection of the analyte was performed at a wavelength of 233 nm using a Waters 2996 photodiode array detector (Waters, Milford, USA) and at a chromatographic retention time of 4.5 min. The method was found to be linear and precise within the range of 1–90 μg/ml. The results of HPLC measurements were used to assess the model’s prediction values.

## Results And Discussion

### API-Substrate Screening

**Figure 3:**
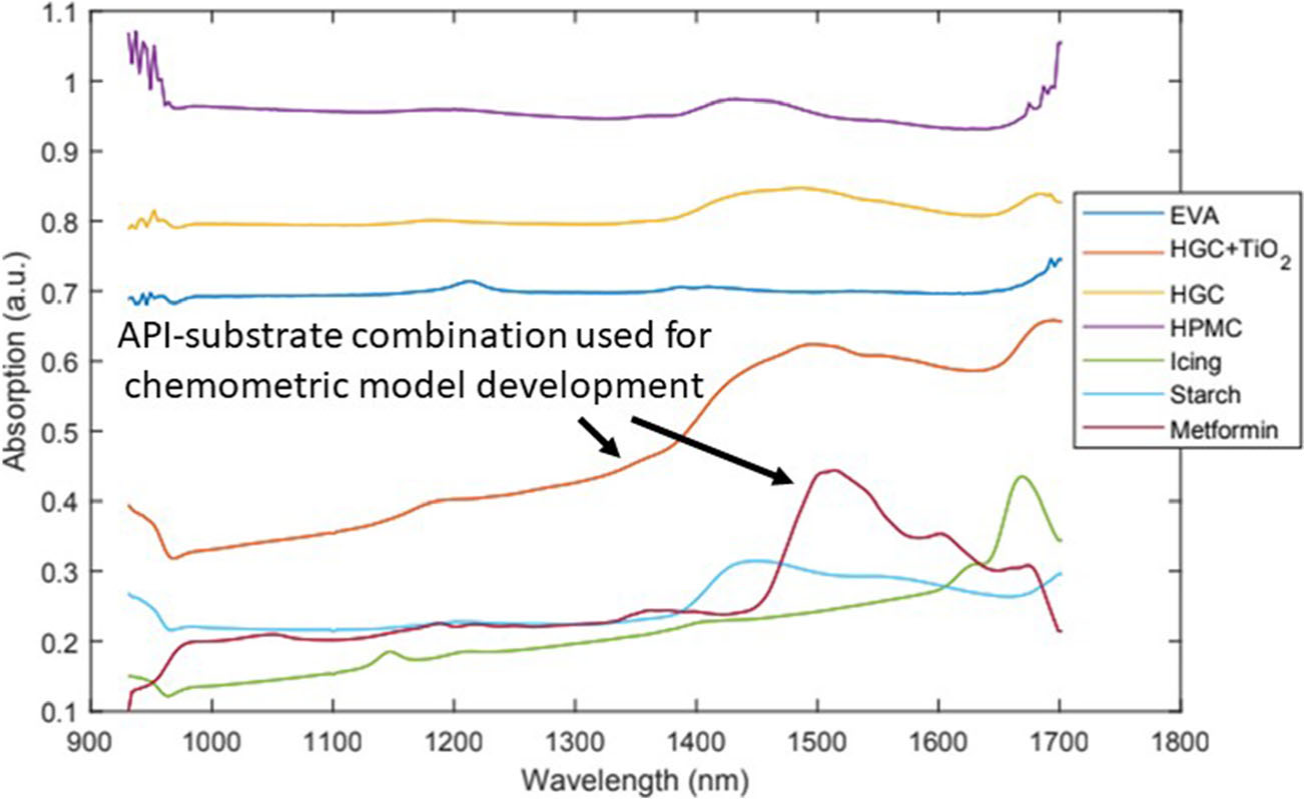
Overlay of individual NIR absorption spectra of all substrates and metformin hydrochloride

In order to screen viable API-substrate combinations, all six substrates were printed with metformin hydrochloride using the patterns described in Section “Printing Pattern for Calibration and Test Samples.” Figure 3 illustrates the corresponding absorption spectra for all pure substrates and metformin hydrochloride. Due to the best performance in terms of clearly distinguishable spectra and handling considerations, gelatin film strips with 2% titanium dioxide (*HGC+TiO*_*2*_) were selected for chemometric model development.

Several aspects were taken into account to evaluate the selected substrate materials regarding their suitability for printing. Taking only the spectral absorption and spectral features into account, *HGC*, *starch*, *icing*, and *HGC+TiO*_*2*_ were the best candidates. The *icing* and *starch* exhibited low spectral absorption, and the spectral features of *starch* and *HGC* were weak. However, *starch and HGC* are highly porous materials thus loosening their structural integrity altering their surface upon contact with the water-based ink. Both aspects can negate the performance of quantitative reflectance spectroscopy. For the *icing* substrate, a limiting factor can be the strong migration of ink into the sugar matrix and a pronounced “coffee-ring effect” (11), both leading to problems for the quantitative spectral imaging of the analyte. HGC+TiO_2_ was found to be the best candidate, as it shows well-distinguishable spectral response in contrast to the API, has low absorption, gives the most defined printing patterns, and has no constraints regarding ink migrating into the carrier matrix.

### Chemometric Model Development

During model development, several attempts were made to improve the prediction performance. As a consequence, all spectra were subjected to various treatments in the final model: standard normal variate (SNV) for scatter correction using a restricted wavelength range of 1200–1600 nm and Savitzky-Golay filter for smoothing and derivation of the spectra, as discussed in Rinnan et al. (33). Savitzky-Golay filtering was accomplished via a second-order polynomial fit with a window size of 35. In order to create a suitable response matrix that reflects the actual distribution of API in the printed drops (comprising of multiple single drops, as described in Section “Printing Pattern for Calibration and Test Samples” and shown in Figure 1a), Raman measurements as discussed in Section “Raman Chemical Imaging” were used, to stratify the training set into regions with different API concentration, before training the models. These Raman measurements revealed a higher concentration in the drop’s center, with a rapid decrease towards its border. This was considered during the model development.

**Figure 4:**
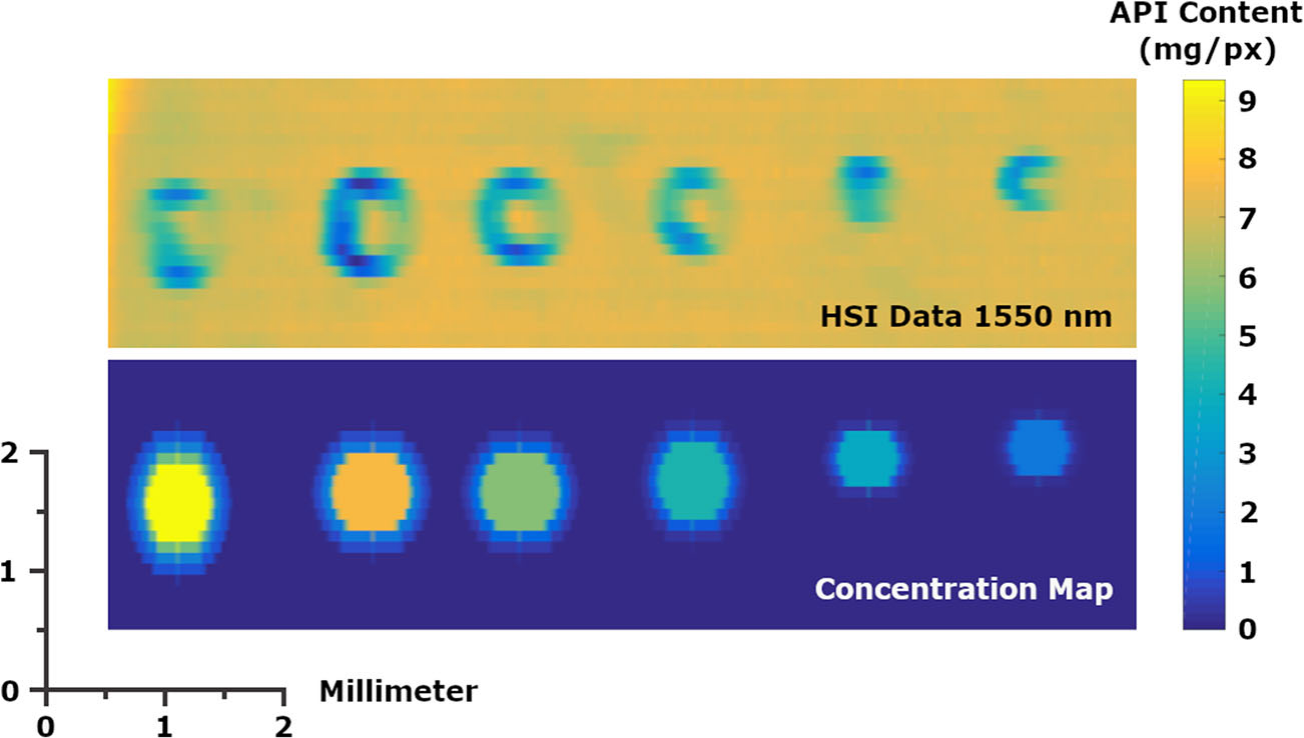
Preprocessed HSI data based on signal intensity at 1515 nm (top) and the created concentration map with colors representing the mass of API in mg/px (bottom)

**Figure 5:**
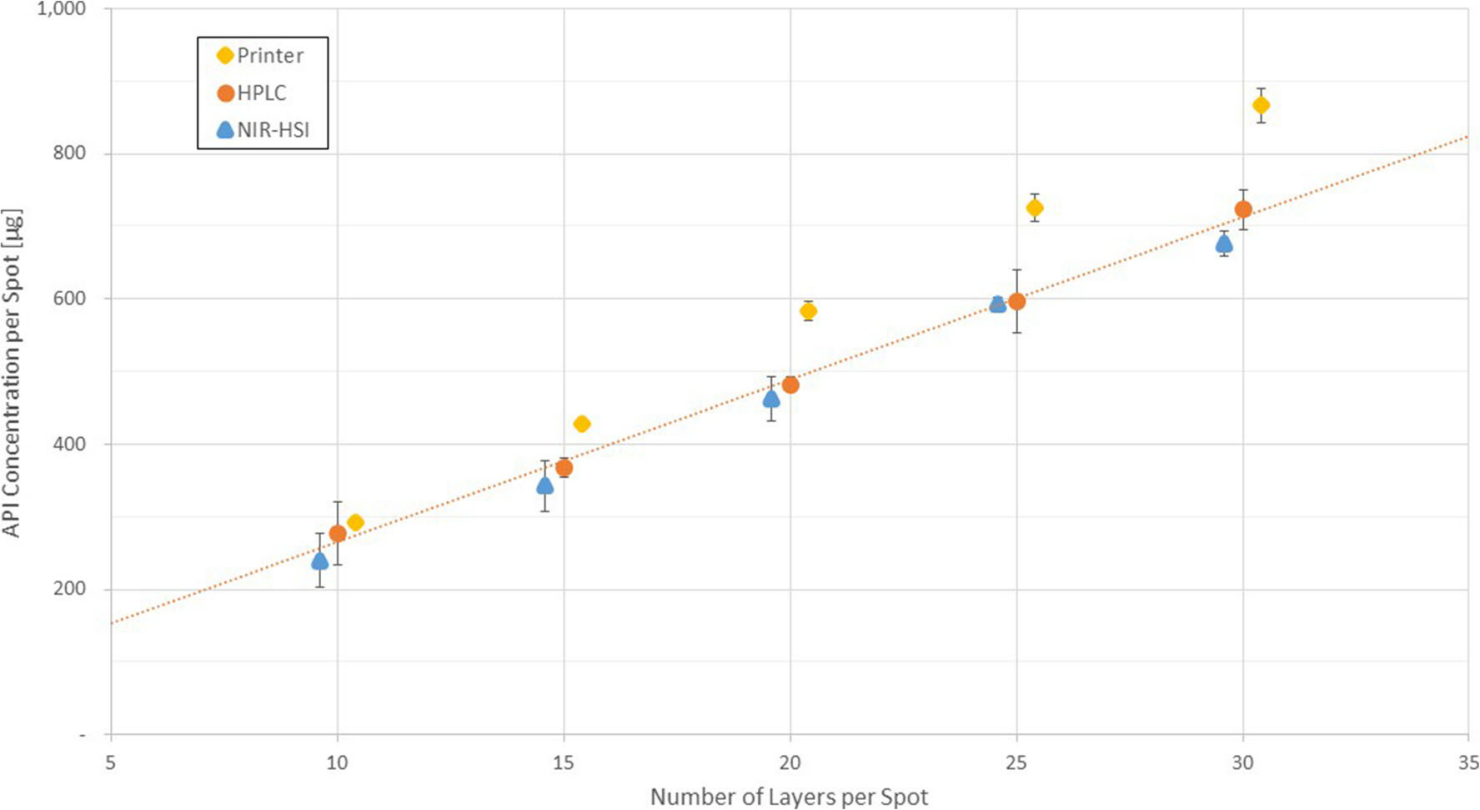
API mass predicted by the NIR-HSI model compared to printer data and HPLC data (reference) of printed test samples. Error bars represent ± one standard deviation (*n* = 3).

Figure 4 illustrates the difference between the preprocessed HSI data raw intensities at the peak absorption at 1515 nm (top) and the calculated API distribution displayed as a heat map (bottom). Since HPLC as a reference method was only applied to spots down to 50 drops, as a consequence of the limit of quantification of the reference method, the spots below were discarded. Iterative improvement of models was undertaken via a back projection approach, comparing the fitted and observed (response) data. The prediction was performed by applying model regression coefficients (beta coefficients) to the preprocessed dataset using multiplication. In the end, a model consisting of 5 principal components was chosen. Comparison with the HPLC data for the measured spots indicated a good correlation between prediction and the reference measurements (HPLC) as illustrated in Figure 5.

### Application of the Developed Model to Predict the Drug Content in Printed Formulations

The final model was applied to printed square-shaped test patterns, as described in Section “Printing Pattern for Calibration and Test Samples” and depicted in Figure 1b. Various test samples were printed, with each test pattern consisting of four printed squares. For pre-validation purpose, predicted mass of API in the individual pixels within each printed square was summarized and compared to printer data (calculated dispensed volume) for the dispensed API within each square and the HPLC data for all test samples as well. Results for the model and the reference method are summarized in Figure 5.

NIR-HSI modeling exhibits a better correlation with HPLC data at low concentrations, which is expected since very thin layers of API can be sampled by the probing light completely and the resulting spectra represent the full volume of interest. With a higher number of layers printed over each other, signal attenuation due to higher needed sampling depths of the probing light is of increasing concern. Another concern with dispensing a high number of droplets on the substrate is the risk of swelling and migration of the ink into sub-surface regions of the carrier matrix. Both mechanisms affect the spot geometry and the expected release profile, which should be considered in future developments for NIR-HSI applications for printed dosage forms as well.

Interestingly, the predicted mass of API, calculated based on the printer data deviates significantly from the NIR-HSI and HPLC data. Since HPLC is considered as the gold standard for determining the amount of API in the pharmaceutical industry, we are tempted to rely on this data and conclude that there is considerable variation in the dispensed drop volume from the print head. In this study, both HPLC and NIR-HSI methods were pre-validated and assessed regarding their suitability for a full method validation. The latter is certainly required for the quality monitoring/control application of the proposed NIR-HSI in industrial printing process and thus can be a part of a follow-up study (34).

**Figure 6:**
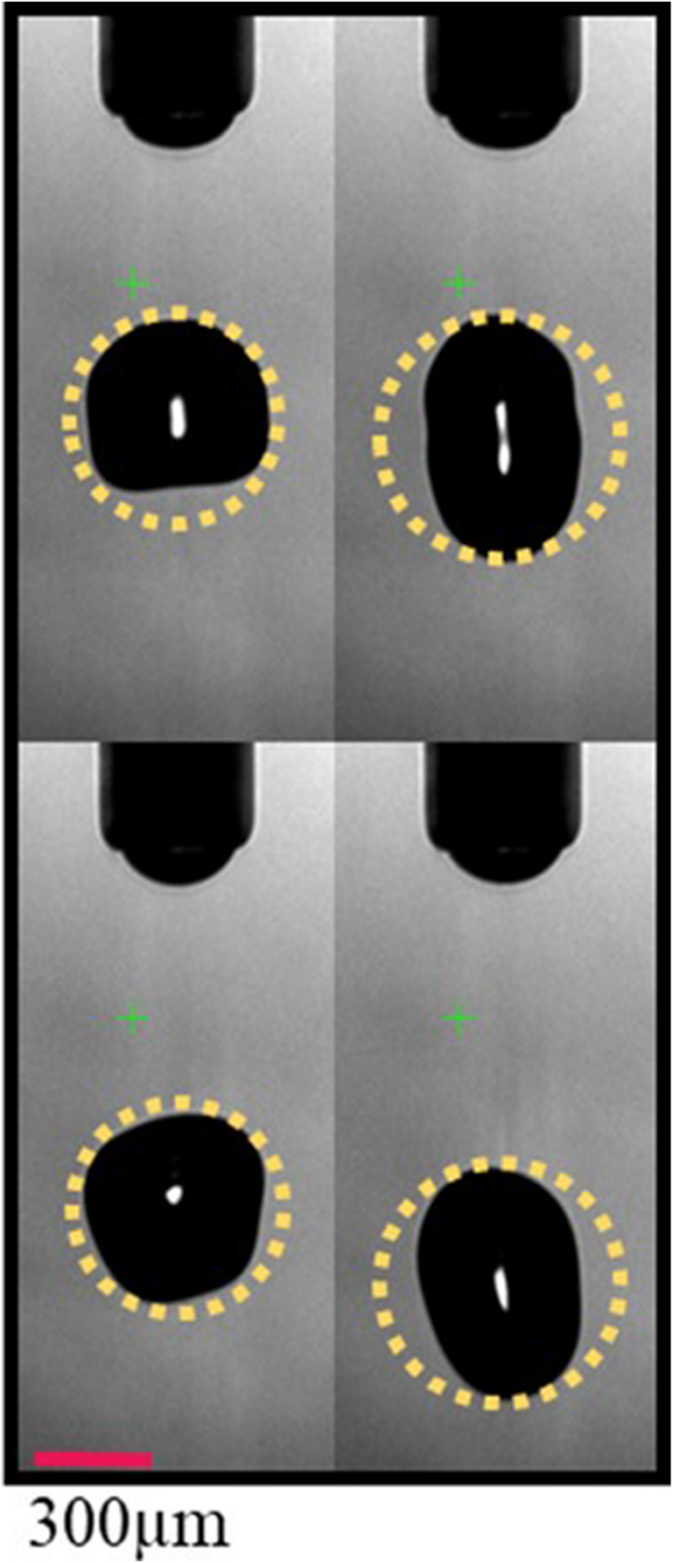
In-line images of dispensed printing solution used for calculation the drop volume. The system calculates drop volumes based on the assumption of perfectly spherical droplets, indicated by the yellow dotted circles

Possible reasons for deviations between printer data, NIR-HSI, and HPLC data could be partial clogging of the printing nozzles and an error introduced by deriving the dispensed volume from 2D images during the initial printer setup assuming perfectly spherical droplets, which is in fact not the case. The latter is described in more detail in Planchette et al. (35) and exemplified in Figure 6. Calculation of dispensed drop volumes by a by circle fit of the drop’s side view (2D projection of a 3D object) is prone to a notable uncertainty as it ignores any non-sphericity of the drops and may be additionally biased by the respective image segmentation algorithm.

Further, looking on the relative standard deviations (RSDs) of the results, it is obvious that the printer data shows nearly constant RSDs in a range of 1.9 to 2.7% for all printed patterns. This is not surprising since the inspection system of the printer only evaluates single droplets (several hundreds of them in some cases) and summarizes the count, i.e., the RSD is nearly independent from the actual number of droplets printed per pattern. For NIR-HSI and HPLC, the situation is somehow different, because RSDs are dominantly affected by the methods’ linearity and S/N ratios. Nevertheless, both methods did not show any deviations during pre-validation within the investigated concentration range with regard to linearity or S/N ratio. Therefore, the elevated RSDs for the low concentrations seem to be introduced by the printer. As a consequence, the RSDs from HPLC and NIR-HSI match with similar RSDs throughout the measurements with high RSD for low concentration (ca. 15% RSD for both methods) and lower RSD for high concentrations (2.5 to 3.8 % RSD)

Other aspects to be considered in 2D printing, and may have contributed to deviations of results, are that physical properties of the ink. The latter needs to be well defined and has to meet the print head requirement precisely. Print heads are highly complex systems with elevated demands on the adherence to the ideal viscosity, density, and surface tension of the ink and with regard to the used dispensing mechanism (Piezo, MEMS, thermo-jet, etc.). These possess quite narrow operational tolerance limits for each print head with regard to the Ohnesorge and Reynolds numbers for the ink used (3, 36). Moreover, accuracy and precision of industrial printing systems are generally specified by the manufacturer, but without reference to the volume of the drops dispensed, the process parameters to be used, and the used jet frequency. The ambient temperature during printing is a critical aspect as well (the required illumination for NIR-HSI can introduce some heat into the setup), as there is a direct influence on surface tension and viscosity of the used inks. These aspects are not that critical for the decorative printing. However, for the quantitative release of active ingredients, they are very critical. Further factors influencing inkjet printing performance include satellite drops and the coffee-ring effect, i.e., phenomena that have gained attention by ongoing academic and industrial research, as outlined in a comprehensive review by Scarpa et al. (11).

Practical considerations for on-demand drug printing of pharmaceutical products which further need to be taken into account are a valid QC strategy (i.e., quality control systems implemented at the site of manufacturing), cleanability of the equipment and cleaning-validation, as well as storage, preservation, stability, and handling of the used inks. The work of Edinger et al. (37), for example, demonstrates the potential combination of 2D inkjet printing and the Quick-Response code (QR) technology to produce dosage forms with important information for the patient and/or healthcare professionals encoded in the dosage form itself. Moreover, a clear definition of non-destructive methods for QC and the availability of printers suitable for the use in a pharmacy setting, along with an introduction of a regulatory path that permits the development and use of personalized medical products, are required for future developments (9). Real-time monitoring of printed dosage forms is another aspect, especially also in the context of continuous manufacturing applications, towards more reliable and traceable pharmaceutical inkjet printing. In our study, we demonstrated one possible route to set up such a system in a way that it will work spatially and temporally resolved, even reporting distribution of deviations in real-time. All of the abovementioned aspects will, to a certain extent, contribute to the future of 2D inkjet printing for pharmaceutical dosage form production.

## Conclusion

This work demonstrates that hyperspectral imaging can be a powerful method for in-line screening of pharmaceutical API-substrate combinations for inkjet printing of water-based inks carrying water-soluble drugs (which are especially challenging due to unfavorable properties with regards to surface tension and density of such systems). Considerations on substrate/ink combinations have been discussed, taking into account not only spectral features but practical aspects of functional printing. Specifically, the developed models facilitate a quantitative visualization of the API distribution as a tool for monitoring the homogeneity of API printed on substrates, e.g., in personalized medicine, enabling easy dosing for clinical trials with individual dosing requirements and on-demand manufactured dosage forms. Further, the developed models offer an opportunity for real-time screening and risk assessment during the development of new printing formulations. Advantages, challenges, and limitations for 2D printing of solid dosage forms have been discussed in the context of on-demand, real-time monitoring of such printing approaches.

Ultimately, the proposed approach could be easily utilized as an on- or even in-line process analytical technology (PAT) tool to measure the dispensed API content after each printing step, i.e., each printed layer, and act as a continuous feedback process control to ensure the final product’s quality. This combination could drastically improve the production efficiency by allowing real-time release testing (RTRT) in on-demand generation of dosage forms for clinical trials or alternatively in local pharmacies or in future industrial continuous inkjet printing lines. One issue that was not addressed in this proof-of-concept study is the influence of drying periods between subsequent printing layers on the results. Thus, key aspects of the future studies are the investigation of the effect of drying and proper positioning of the subsequent NIR-monitoring step.
